# The puzzling interplay between p53 and Sp1

**DOI:** 10.18632/aging.101238

**Published:** 2017-05-09

**Authors:** Ariella Oppenheim, Galit Lahav

**Affiliations:** Department of Hematology, Hebrew University Faculty of Medicine, Jerusalem 91120, Israel

**Keywords:** cancer, p53, Sp1, transcription factors, apoptosis, proliferation

The paradigm tumor suppressor protein p53 was initially discovered in the Seventies by a number of investigators as a protein that was tightly bound to SV40 large T-antigen, a “contaminant” during T-antigen purification [1]. Its role as a tumor suppressor was realized only a few years later, when it was found that it is absent or mutated in many tumors. Since then p53 has been described as the “guardian of the genome”, due to its central role in keeping genome integrity in response to genotoxic stress as well as many other insults [2].

Specificity Factor 1, Sp1, was the first mammalian transcription factor to be purified and characterized by Dynan and Tjian in the early Eighties. Sp1 is overexpressed in many types of cancer, including those carrying wild-type p53. Sp1 therefore is considered as a ‘hallmark of cancer’ and a candidate target for cancer therapy [3].

p53 is activated in response to stress [2] and activates or represses a variety of target pathways involved in DNA repair, cell‐cycle arrest, senescence, or apoptosis [1]. It recognizes thousands of binding sites in the human genome (Li et al, 2014). The choice of p53 target genes depends on a number of parameters including p53 protein levels, its temporal pulsing behavior [4], post-translational modifications [5], and interaction with other co-factors.

Like p53, Sp1 regulatory activity is also dependent on its levels, post translation modifications and interactions with other factors [6]. And like p53, sp1 also participates in regulation of cell growth, development, and intriguingly also in apoptosis [7].

Both p53 and Sp1 therefore are central cellular transcription factors, regulating critical cellular life and death decisions. The complex interactions between them are just beginning to be unfolded. Intriguingly, p53 and Sp1 share similar consensus sequences at GC-boxes along the human genome, suggesting that they might interplay in transcription regulation and may even compete in binding to specific promoters or function in opposite directions. For example, both transcription factors bind to the GC-box region at the promoter of the SV40 T-antigen. T-antigen (tumor-antigen) promotes cellular immortalization, facilitating tumorogenesis by other factors. As would be expected from an oncogene, Sp1 is required to activate T-antigen expression. On the other hand the tumor suppressor p53 was recently found to repress T-antigen transcription, preventing SV40 propagation in p53 expressing cells, thus functioning in host defense against the Infecting virus [8].

The levels of p53 protein can be elevated by reagents that interfere with the action of its negative regulator Mdm2. For example, Nutlin3 increases p53 levels by binding to Mdm2 and inhibiting its association with p53 while RITA activates p53 by binding to the protein itself, preventing binding of Mdm2. Recently Sp1 was also found to be regulated by Nutlin, but in a different manner. Nutlin treatment was observed to reduce Sp1 level, presumably by leading it to Mdm2-mediated proteosomal degradation [7].

A recent comprehensive ChIP-seq study [7] discovered additional complex interactions between p53 and Sp1 in gene regulation. Hundreds of p53 target genes were found to contain conserved Sp1 response nts, in the vicinity (±500 bp) of p53 binding sites. This finding suggested co-regulation by both factors. Indeed both Sp1-depletion and Sp1 ectopic expression had a profound effect on about half of the p53 regulated genes. In some of the genes the effect of p53 changed from repression to induction, while in others it had the opposite effect. Furthermore, it was found that both transcription factors enhance the binding of one another to DNA. The data further indicated that activation of p53 by RITA, but not by Nutlin3, significantly increase the binding of Sp1, suggesting recruitment of Sp1 by RITA-activated p53. Remarkably, these findings demonstrated that Sp1 was required for the induction of p53-dependent pro-apoptotic pathways participating in cancer and in apoptosis, in particular the MAPK and Wnt signaling pathways. Intriguingly, in contradiction to the presumed function of Sp1 as an oncogene, this study demonstrated that Sp1 is a crucial factor for robust p53-mediated apoptosis but not cell cycle arrest [7]. The central transcription factors p53 and Sp1, as well as their binding to similar DNA elements, were discovered decades ago. Nevertheless their mutual effects in complex cellular regulation, including life and death decision are just beginning to unravel. New technologies looking at the combined and separated functions of these transcription factors under various genetic background will accelerate research on their multifaceted interactions and is promised to facilitate drug discovery with a particular focus for cancer therapy.
